# Supplementary material for: Organization of an Ascending Circuit that Conveys Flight Motor State
Source: bioRxiv. 2023 Jun 9:2023.06.07.544074. Preprint. [Version 1] doi: 10.1101/2023.06.07.544074 (PMC10274802; doi:10.1101/2023.06.07.544074)
Supplement: Supplement 1 [file NIHPP2023.06.07.544074v1-supplement-1.pdf]

bioRxiv preprint doi: https://doi.org/10.1101/2023.06.07.544074; this version posted June 9, 2023. The copyright holder for this preprint (which was not certified by peer review) is the author/funder, who has granted bioRxiv a license to display the preprint in perpetuity. It is made available under aCC-BY 4.0 International license.

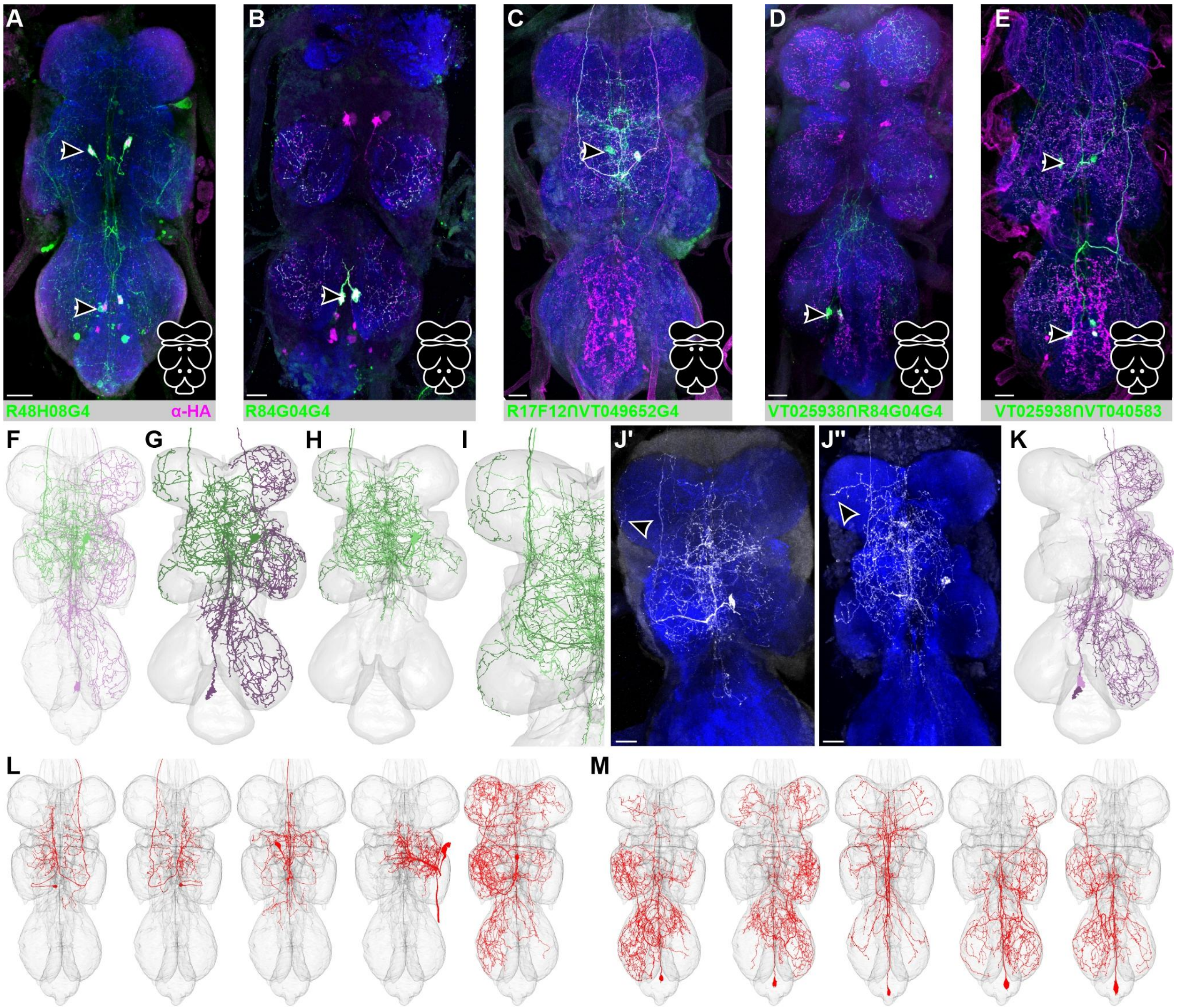

Figure S1. Validation of driver lines including the AHNs driving expression of GFP (green) and immunolabeled for histamine (magenta). NCAD is used as a neuropil marker (dark blue). Cartoon schematic at lower right corner indicates which AHN pairs are expressed by a given driver line. **A)** R48H10-Gal4 (both AHN pairs included). **B)** R84G04-Gal (MtAHNs included). **C)** R17F12 ∩ VT049652 splitGal4 (MsAHN only). **D)** VT025938 ∩ R84G04 splitGal4 (MtAHNs only). **E)** VT025938 ∩ VT049652 splitGal4 (both AHN pairs). **F-G)** reconstructions of the MsAHN (green) and MtAHN (magenta) in the **F)** MANC and **G)** FANC datasets. **H)** Overlaid reconstructions of a single MsAHN from the MANC (light green) and FANC (dark green) datasets. **I)** The processes of MsAHNs within the prothoracic neuromere differ between MANC and FANC in terms of the extent to which they project laterally. **J)** Single MCFO clones of a male MsAHN showing the variability in the prothoracic branching consistent with differences between FANC and MANC. **K)** Overlaid reconstructions of a single MtAHN from the MANC (light

bioRxiv preprint doi: https://doi.org/10.1101/2023.06.07.544074; this version posted June 9, 2023. The copyright holder for this preprint (which was not certified by peer review) is the author/funder, who has granted bioRxiv a license to display the preprint in perpetuity. It is made available under aCC-BY 4.0 International license.

magenta) and FANC (dark magenta) datasets revealed no obvious morphological differences. **L)** Top 5 non-redundant NBLAST hits from all neurons with soma in T2 in the MANC dataset queried against the pair of MsAHN candidates. Body IDs from left to right are 11628, 12486, 10378, 10178 and 10222. **M)** Top 5 NBLAST hits from all neurons with soma in T3 in the MANC dataset queried against the pair of MtAHN candidates. Body IDs from left to right are 10197, 10330, 10066, 10104 and 10109. All scale bars = 20μm.

bioRxiv preprint doi: https://doi.org/10.1101/2023.06.07.544074; this version posted June 9, 2023. The copyright holder for this preprint (which was not certified by peer review) is the author/funder, who has granted bioRxiv a license to display the preprint in perpetuity. It is made available under aCC-BY 4.0 International license.

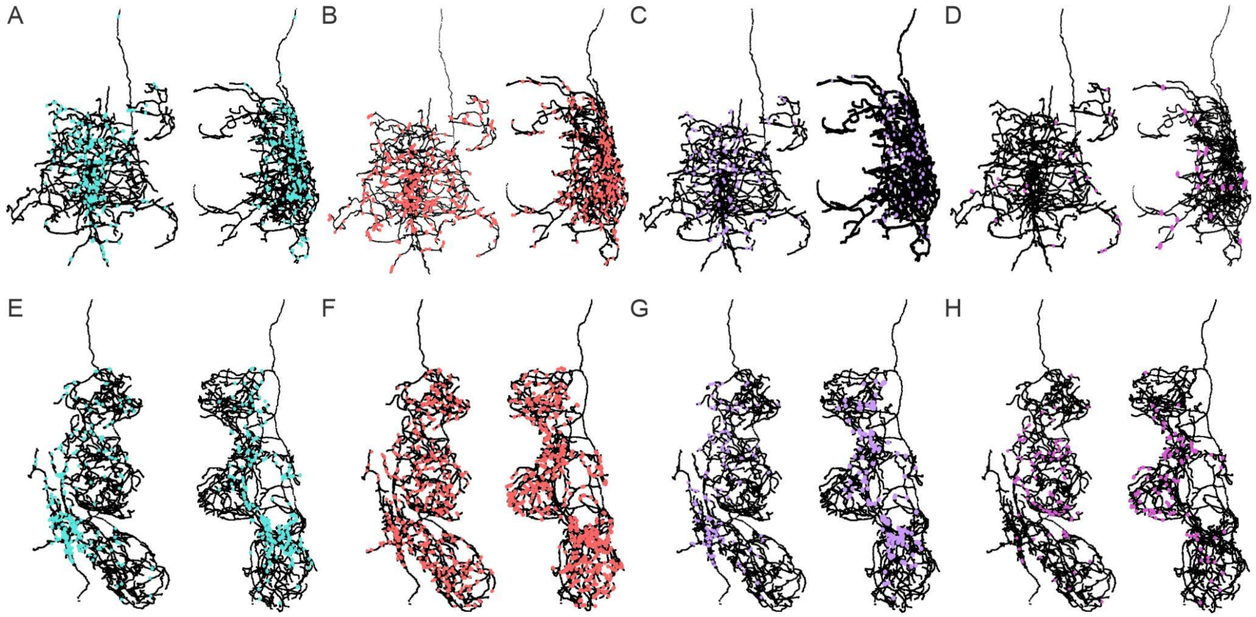

Figure S2. Input synapse distribution on AHNs by cell class. A-D) Horizontal (left) and sagittal (right) views of the synapse distributions upon the left MsAHN from **A)** descending neurons (cyan), **B)** interneurons (red), **C)** ascending neurons (lavender) and **D)** sensory neurons (pink). **E-H)** Horizontal (left) and sagittal (right) views of the synapse distributions upon the left MtAHN from **E)** descending neurons (cyan), **F)** interneurons (red), **G)** ascending neurons (lavender) and **H)** sensory neurons (pink).

bioRxiv preprint doi: https://doi.org/10.1101/2023.06.07.544074; this version posted June 9, 2023. The copyright holder for this preprint (which was not certified by peer review) is the author/funder, who has granted bioRxiv a license to display the preprint in perpetuity. It is made available under aCC-BY 4.0 International license.

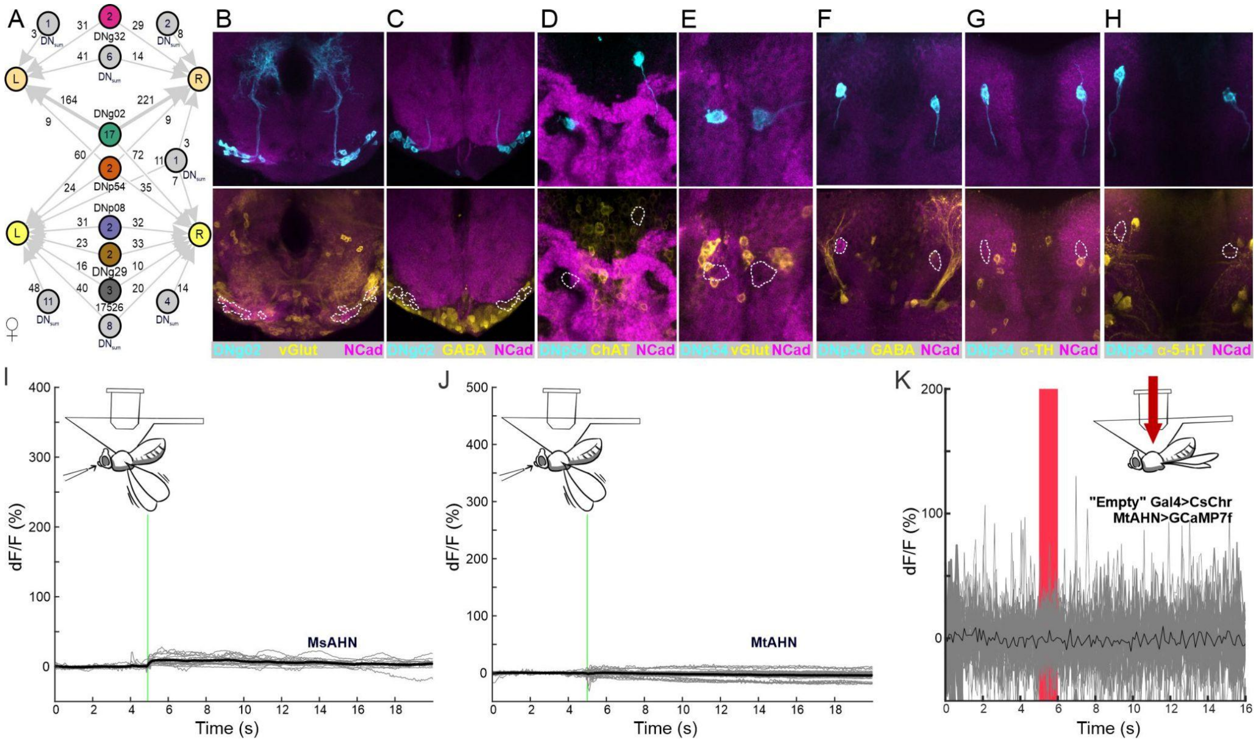

Figure S3. **DN input to AHNs in FANC, DN neurotransmitter usage, and AHN calcium imaging controls. A)** Graph plot of upstream DNs to the MsAHNs and MtAHNs in the FANC dataset. **B)** Intersection between the DNg02 splitGal4 (cyan) and a vGlut-T2A-LexA (yellow) driver line reveals that the DNg02s are not glutamatergic. **C)** The DNg02s (cyan) does not immunolabel for GABA (yellow). **D-H)** The DNp54 splitGal4 line (cyan) does not overlap with T2A-LexAs for **D)** ChAT (yellow) or **E)** vGlut (yellow), nor do the DNp54s immunolabel for **F)** GABA (yellow), **G)** tyrosine hydroxylase (TH; yellow) or **H)** serotonin (5-HT; yellow). NCAD (magenta) delineates neuropil. **I-J**) Flight-induced changes in fluorescence due to movement measured via GFP expression in **I**) the MsAHNs (6 flies, 6 soma, 3 trials) and **J)** the MtAHNs (9 flies, 13 soma, 3 trials). Cartoon depicts orientation of flies during each recording and green line indicates timing of an air puff to trigger flight. Gray traces represent recordings from individual AHN soma and black trace represents the average fluorescence transient across all animals. **K)** Ca^{2+} transients evoked from the MtAHNs in response to CsChrimson activation of an "empty" Gal4 line. Gray traces represent recordings from individual AHN soma and black trace represents the average fluorescence transient across all animals.

bioRxiv preprint doi: https://doi.org/10.1101/2023.06.07.544074; this version posted June 9, 2023. The copyright holder for this preprint (which was not certified by peer review) is the author/funder, who has granted bioRxiv a license to display the preprint in perpetuity. It is made available under aCC-BY 4.0 International license.

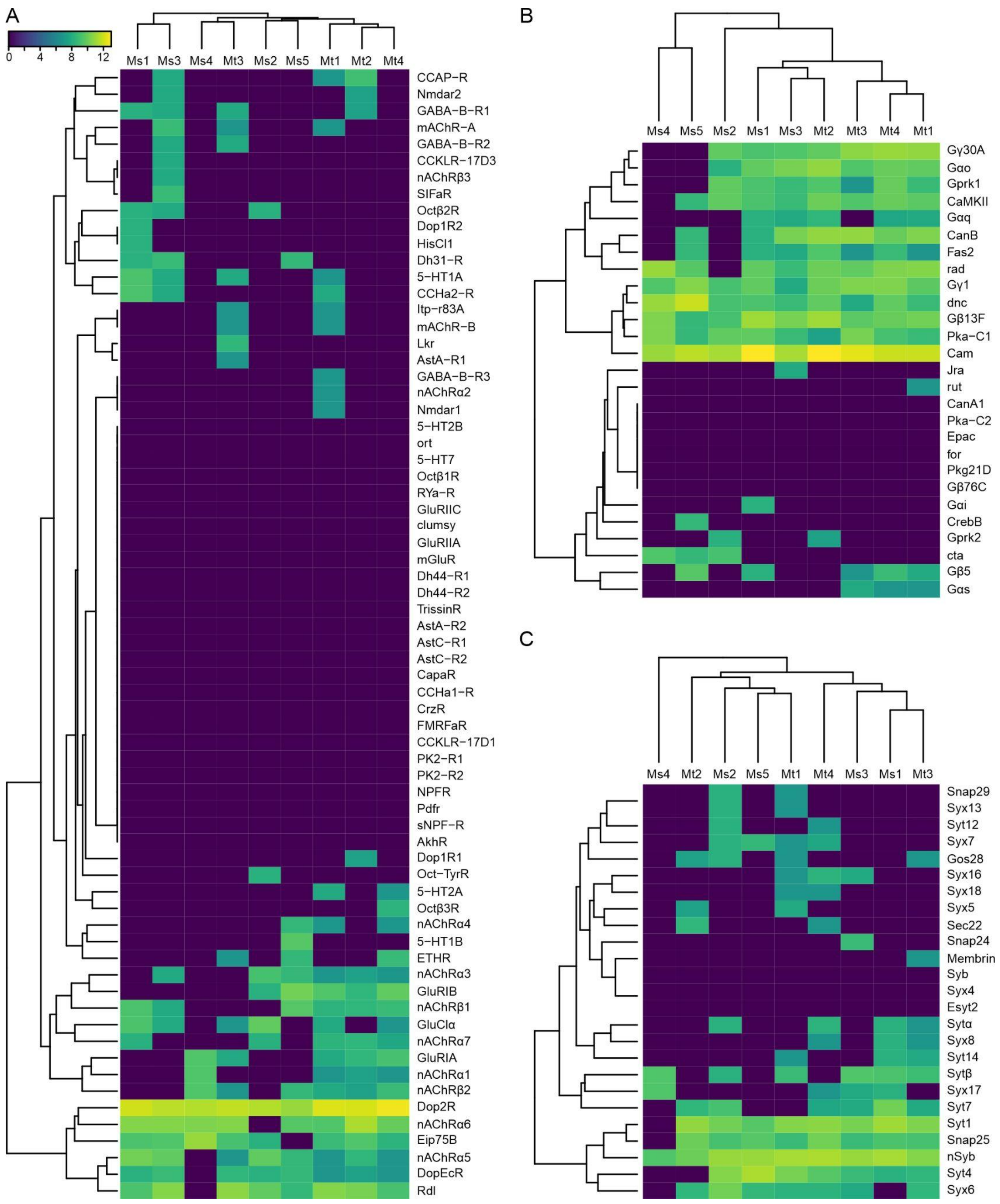

Figure S4. **AHN expression profiling. A)** Hierarchically-clustered heatmap showing normalized expression of neurotransmitter receptor genes screened in cluster analysis. **B)** Hierarchical cluster analysis showing normalized 2nd messenger associated gene product

bioRxiv preprint doi: https://doi.org/10.1101/2023.06.07.544074; this version posted June 9, 2023. The copyright holder for this preprint (which was not certified by peer review) is the author/funder, who has granted bioRxiv a license to display the preprint in perpetuity. It is made available under aCC-BY 4.0 International license.

expression across candidate AHNs. **C)** Hierarchical cluster analysis showing normalized expression of synaptic vesicle transmission genes across AHN candidates. Values are read counts of each gene normalized by total counts per million (CPM) per cell, then log scaled (log2(n+1)).

bioRxiv preprint doi: https://doi.org/10.1101/2023.06.07.544074; this version posted June 9, 2023. The copyright holder for this preprint (which was not certified by peer review) is the author/funder, who has granted bioRxiv a license to display the preprint in perpetuity. It is made available under aCC-BY 4.0 International license.

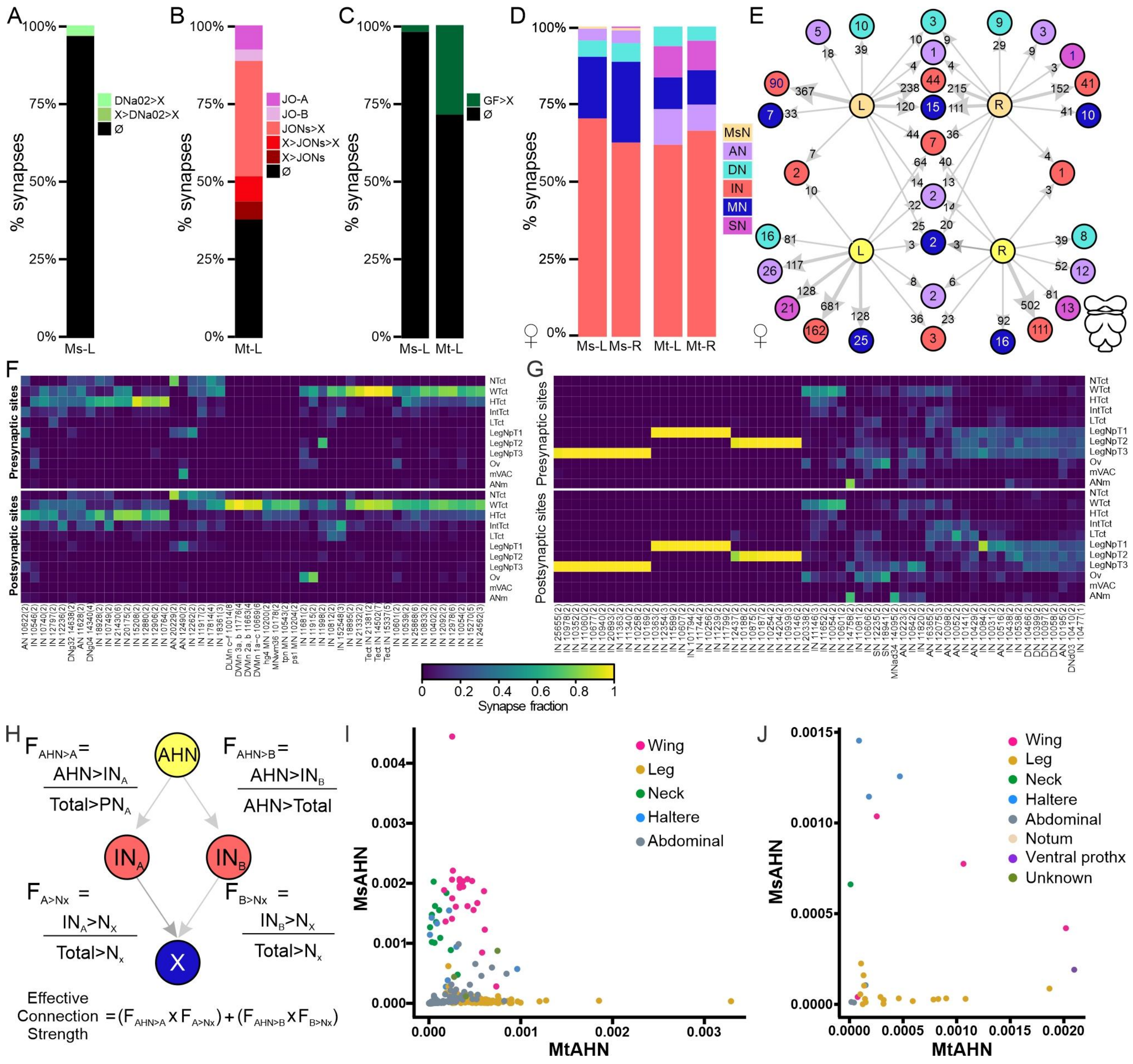

Figure S5. **Further information about AHN downstream partners. A)** Synapse fractions for MsAHN downstream partners in FAFB highlighting those with the DNa02s whether it be upstream ("X>DNa02") or reciprocal ("X>DNa02>X") connectivity. "∅" indicates neurons with no connectivity to DNa02. **B)** Synapse fraction for the downstream partners of the MtAHN in FAFB classified based on JON type, or connectivity to the JONs otherwise. "X>JONs" indicates neurons upstream of the JONs, "X>JONs>X" indicates neurons both up and downstream of the JONs (threshold of 2 synapses), and "JONs>X" indicates neurons downstream of the JONs. Connectivity of AHN downstream cells with JONs were stochastically traced, and cell count connected to JONs are likely underestimated. **C)** Synapse fractions for MsAHN and MtAHN downstream partners in FAFB that are upstream from from the Giant Fiber neurons ("X>GF").

bioRxiv preprint doi: https://doi.org/10.1101/2023.06.07.544074; this version posted June 9, 2023. The copyright holder for this preprint (which was not certified by peer review) is the author/funder, who has granted bioRxiv a license to display the preprint in perpetuity. It is made available under aCC-BY 4.0 International license.

D) Synapse fractions for the downstream partners of the MsAHNs and MtAHNs from the FANC dataset. **E)** Graph plot for the downstream partners of the MsAHNs (peach) and MtAHNs (yellow) from the FANC dataset. **F-G)** Normalized proportion of presynaptic or postsynaptic sites of downstream targets of the **F)** MsAHNs and **G)** MtAHNs plotted based on VNC neuropil within the MANC dataset. Only downstream partners of groups above an outlier threshold of synapse counts with AHNs (above the 3rd quartile plus 1.5 x interquartile range) were included. Neuropil included were based on neck tectulum (NTct), wing tectulum (WTct), haltere tectulum (HTct), intermediate tectulum (IntTct), lower tectulum (LTct), prothoracic leg neuropil (LegNp.T1), mesothoracic leg neuropil (LegNp.T2), metathoracic leg neuropil (LegNp.T3), ovoid/accessory mesothoracic neuropil (Ov), medial ventral association center (mVAC), abdominal neuromeres (ANm). **H)** Cartoon representation of effective connection strength calculations used in Figure 6J and K. $F_{AHN>A}$ is the proportion of the total synapses to a given interneuron (“IN_A”) that are provided by a given AHN, and $F_{A>Nx}$ is the proportion of the total synapse to a given downstream neuron (“N_x”) provided by IN_A. The effective connection strength is therefore the sum of the products of $F_{AHN>A}$ and $F_{A>Nx}$ for every downstream interneuron that is upstream of Nx. **I-J)** Scatterplot of the adjacency scores for all **I)** motor neurons or **j)** sensory neurons within the VNC relative to both AHN pairs. Color-coding is based on body part; wing (pink), leg (gold), neck (green), haltere (blue), abdominal (grey), notum (pink), ventral prothorax and unknown (dark green).

bioRxiv preprint doi: https://doi.org/10.1101/2023.06.07.544074; this version posted June 9, 2023. The copyright holder for this preprint (which was not certified by peer review) is the author/funder, who has granted bioRxiv a license to display the preprint in perpetuity. It is made available under aCC-BY 4.0 International license.

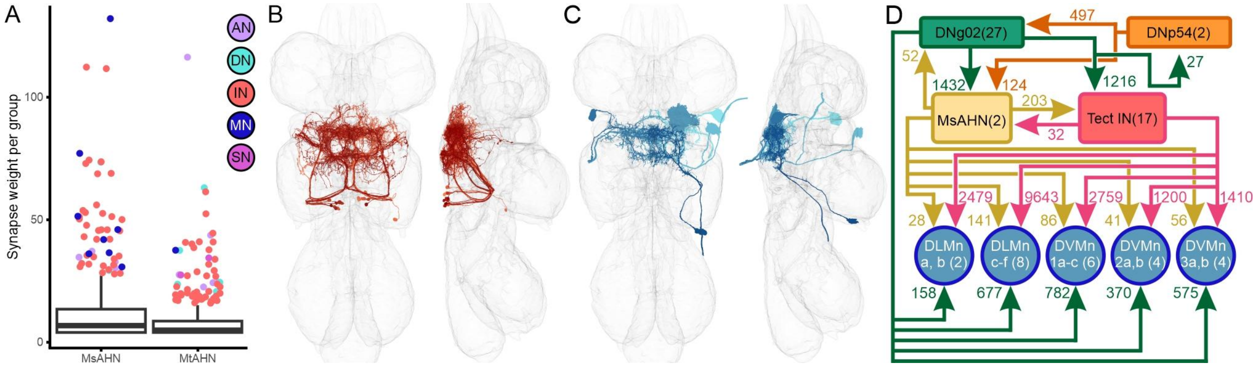

Figure 7. MsAHNs form a feedforward with the DNg02s to directly and indirectly target wing motor neurons. **A)** Box and whisker plot of AHN downstream partners for number of synapses per synaptic partner group (neurons with matching morphology) with the MsAHNs and MtAHNs. Outliers above the 3rd quartile plus 1.5 x interquartile range are plotted individually and color-coded based on cell class. **B-C)** The cell classes receiving the greatest amount of synaptic input from the MsAHNs were **B)** a population of tectular interneurons (shades of red) and **C)** wing motor neurons (shades of blue); only a subset of tectular interneurons and one of each wing motor neuron type are shown for clarity. **D)** Circuit motif depicting the relationship between the DNg02s (green), DNp54s (orange), MsAHNs (yellow), tectular interneurons (red) and wing MNs (blue). The DNg02s and DNp54s are reciprocally connected and both synapse upon the MsAHNs. The DNg02s and MsAHNs synapse upon the tectular interneurons and wing MNs (MNs of the dorsal longitudinal muscles; DLMn a, b and DLMn c-f, and of the dorsal ventral muscles; DVMn 1a-c, DVMn 2a, b and DVMn 3a, b).

bioRxiv preprint doi: https://doi.org/10.1101/2023.06.07.544074; this version posted June 9, 2023. The copyright holder for this preprint (which was not certified by peer review) is the author/funder, who has granted bioRxiv a license to display the preprint in perpetuity. It is made available under aCC-BY 4.0 International license.

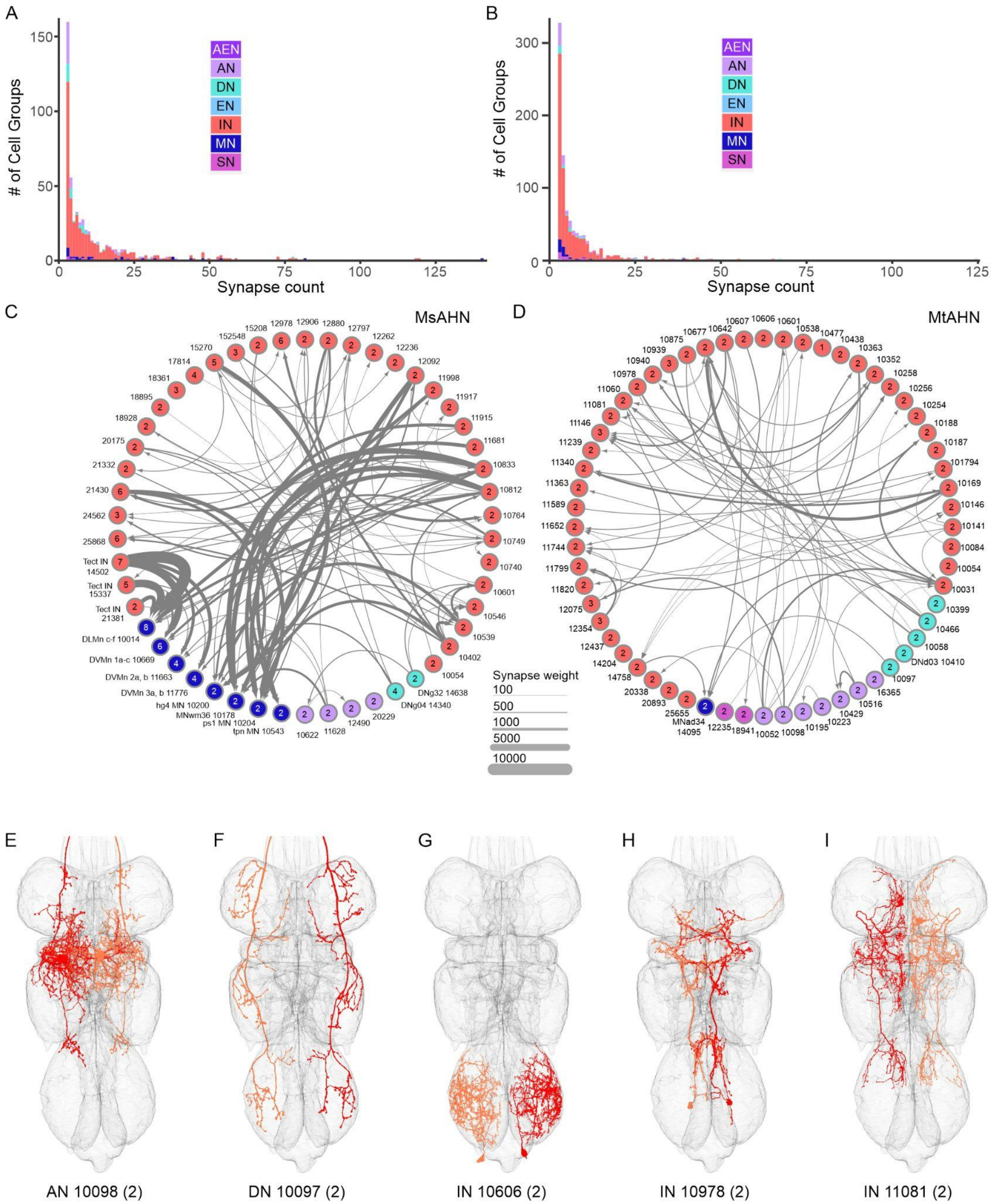

Figure S6. **Synapse count distribution, interconnectivity and morphology of AHN downstream partners. A-B)** Synapse count distribution of **A)** MsAHN and **B)** MtAHN downstream partner groups color coded based on cell-class in MANC. **C-D)** Graph plot depicting the interconnectivity of the downstream partners of **C)** the MsAHNs and **D)** the
